# Reinforcement learning based variable damping control of wearable robotic limbs for maintaining astronaut pose during extravehicular activity

**DOI:** 10.3389/fnbot.2023.1093718

**Published:** 2023-02-15

**Authors:** Sikai Zhao, Tianjiao Zheng, Dongbao Sui, Jie Zhao, Yanhe Zhu

**Affiliations:** State Key Laboratory of Robotics and Systems, Harbin Institute of Technology, Harbin, China

**Keywords:** extravehicular activity, reinforcement learning, wearable robotic limbs, variable damping control, modular robot

## Abstract

As astronauts perform on-orbit servicing of extravehicular activity (EVA) without the help of the space station’s robotic arms, it will be rather difficult and labor-consuming to maintain the appropriate position in case of impact. In order to solve this problem, we propose the development of a wearable robotic limb system for astronaut assistance and a variable damping control method for maintaining the astronaut’s position. The requirements of the astronaut’s impact-resisting ability during EVA were analyzed, including the capabilities of deviation resistance, fast return, oscillation resistance, and accurate return. To meet these needs, the system of the astronaut with robotic limbs was modeled and simplified. In combination with this simplified model and a reinforcement learning algorithm, a variable damping controller for the end of the robotic limb was obtained, which can regulate the dynamic performance of the robot end to resist oscillation after impact. A weightless simulation environment for the astronaut with robotic limbs was constructed. The simulation results demonstrate that the proposed method can meet the recommended requirements for maintaining an astronaut’s position during EVA. No matter how the damping coefficient was set, the fixed damping control method failed to meet all four requirements at the same time. In comparison to the fixed damping control method, the variable damping controller proposed in this paper fully satisfied all the impact-resisting requirements by itself. It could prevent excessive deviation from the original position and was able to achieve a fast return to the starting point. The maximum deviation displacement was reduced by 39.3% and the recovery time was cut by 17.7%. Besides, it also had the ability to prevent reciprocating oscillation and return to the original position accurately.

## 1. Introduction

With the progress of robot technology and artificial intelligence, space exploration has ushered in rapid development (Chien and Wagstaff, 2017; Jacobstein et al., 2017; Lester et al., 2017). The world’s space powers began to carry out manned space activities around the International Space Station (ISS) (Flores-Abad et al., 2014; Jiang et al., 2017; Ruttley et al., 2017). In addition, commercial space tourism has gradually become a new highlight of manned space development in recent years. Some private manned space companies have successfully completed several commercial space trips (Webber, 2013; Chang, 2015). It can be established that manned space engineering will play an increasingly important role in space exploration and on-orbit servicing. Therefore, there will be higher requirements for astronauts’ operations in the outer space environment.

Extravehicular activity (EVA) refers to when astronauts wear spacesuits to perform tasks outside the spacecraft, which is the key technology of manned space engineering. The environment of EVA is extreme, work intensity is high, and the operation process is complex. These issues greatly restrict the astronaut’s EVA time and success rate. In recent years, various robot-intelligent technologies have been applied to assist in the reduction of work intensity and improve workability (Zhang et al., 2020a; Wang S. et al., 2022). However, they have not been popularized and applied in the space field. For on-orbit servicing of EVA, there are two main modes of extravehicular movement. One is that astronauts move in vast space through the robotic arm of the space station (Nokleby, 2007; McHenry et al., 2020). The other is that astronauts climb by themselves with the help of safety ropes. In the former scenario, astronauts’ lower limbs are fixed to the robotic arm, which can provide a foot restrictor and liberate the upper limbs to accomplish tasks. However, there are some areas where the space station’s robotic manipulator cannot reach. In these areas, with the lack of a space robotic manipulator, astronauts will have to move and work by themselves under the protection of safety ropes. When working in this situation, it is significantly hard for them to maintain a suitable position when they suffer some form of impact. In order to maintain stability, they need to exert force with one or two hands, which not only increases energy consumption but also greatly limits work efficiency. Thus, astronauts need additional devices that assist them in resisting impact and maintaining position during the process of EVA. There is much research on trajectory planning and control of robotic manipulators at home and abroad (Zhang et al., 2020b, 2022; Zhao et al., 2022). However, these large dedicated robots and equipment have high launch costs and low utilization rates. Several astronaut-assisting robots have been developed, including humanoid robots (Diftler et al., 2011; Ackerman, 2019), on-orbit modeling robots (Zykov et al., 2007; Post et al., 2021), and wearable-assisting robots (Hall, 2013; Zhao et al., 2021). The primary application purpose of these devices is to provide astronauts with operation or strength enhancement assistance for on-orbit servicing. In addition, they are either still in the conceptual design stage or can only be used inside the cabins of the ISS. Thus, none of them can provide astronauts with the ability to withstand external impact. Although some impact-resisting methods of the space station’s robotic arms have been studied (Su et al., 2020; Liu et al., 2021; Olivieri et al., 2021; Raina et al., 2021; Wang X. et al., 2022), they are only used for the robotic arms themselves or on missions to capture free-flying objects, and not in helping astronauts. In addition, the main problem is that large space manipulators cannot be applied to all task scenes.

Wearable robotic limbs can provide a new method for assisting astronauts in performing tasks, especially those carrying out extravehicular work alone. The robotic limb can act as an extra limb of the astronaut and improve the wearer’s abilities of perception and operation. In this way, it has the potential to reduce the astronaut’s physical exertion and consumption in extravehicular activities and improve the success rate of on-orbit servicing tasks. Considering the safe and comfortable operation requirements of astronauts, the wearable robotic limb system is expected to have the following impact resistance capabilities: (1) Deviation resistance: The deviation after impact cannot be too large. It is very dangerous to deviate too far from the operating position. In case of an emergency, astronauts should have the ability to grasp the handrail; (2) Fast return: After reaching the maximum deviation position, it can quickly return to the initial position. It is helpful to extend the effective working time of EVA; (3) Oscillation resistance: After the system quickly restores to the initial position, it is necessary to prevent reciprocating oscillation relative to the initial position, which will cause system instability and physical discomfort; and (4) Accurate return: The system must be able to return to the original position after impact. Otherwise, astronauts need to make additional manual adjustments, which indirectly increases the difficulty and physical exertion of the task.

As far as we know, no similar concepts of robots for assisting astronauts have been proposed yet. The purpose of this paper is to propose a variable damping control method based on a reinforcement learning algorithm for wearable robotic limbs, in which the virtual damping is trained to be adjustable to meet the impact resistance requirements proposed above. The method was verified in a simulation environment, which ensured that the robotic limb system has the ideal impact-resisting ability. The rest of the paper is organized as follows: Section 2 introduces the basic composition of the wearable robotic limbs for astronauts and explains the variable damping control method based on Reinforcement Learning; Section 3 presents the simulation results and evaluation; and Section 4 summarizes the whole work, analyzes the application limitations, and outlines plans for future work.

## 2. Materials and methods

### 2.1. Wearable robotic limb system

**FIGURE 1:**
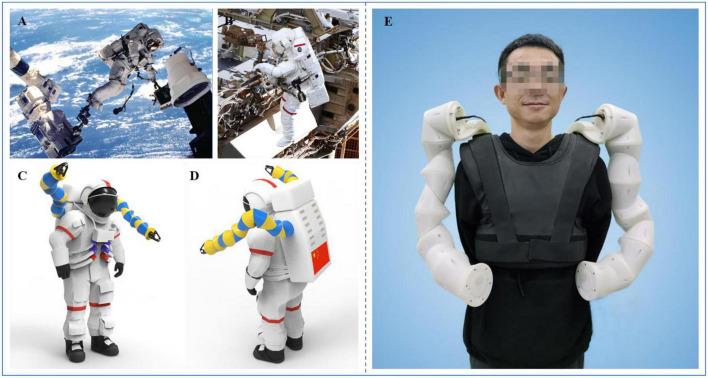
Wearable robotic limbs for astronauts. **(A)** Performing EVA with the help of the space station’s robotic arm (Mohon, 2014). **(B)** Performing EVA without the space station’s robotic arm (Garcia, 2019). **(C)** The rendered view of the front side of the wearable robotic limbs for astronauts. **(D)** The rendered view of the back side of the wearable robotic limbs for astronauts. **(E)** Wearing display of the robotic limbs for astronauts.

Astronauts can work in orbit outside the cabin of the ISS in two main ways. The first is that the astronaut’s feet are attached to the end of the space station’s robotic arm. As shown in Figure 1A, the space station’s robotic arm provides the astronaut with a foot restrictor, so that the astronaut can maintain the desired position through the lower limbs. Meanwhile, the upper limbs and hands are free to perform tasks. The second is that the astronaut is connected to the working area via a safety rope without using the space station’s robotic arm. In this case, there is no reliable anchor point such as the foot restrictor. If the astronaut wants to maintain a proper working position, one hand is needed to maintain that position, as shown in Figure 1B. In this situation, it is not suitable for the astronaut to operate with both hands simultaneously, and the astronaut cannot perform complex operational tasks that call for two-handed cooperation. In addition, it will consume considerable energy and reduce the EVA time.

In view of the above shortcomings, we proposed a wearable robotic limb system that can be fixed onto the astronaut’s backpack as additional arms to assist in moving and operating outside the ISS. The system is named AstroLimbs (Zhao et al., 2021). Figures 1C,D show the rendered views of the front and back sides of the AstroLimbs, respectively. The wearing display of the robotic limb system is shown in Figure 1E. Based on the modular design concept, each robotic limb is composed of six identical basic modules connected in series. The modular design concept is suitable for space engineering, with more convenient assembly, better interchangeability, and improved fault tolerance. The end faces of both submodules are equipped with the connection mechanism. Two basic modular units can be connected in series via the connection mechanism. Each basic module serves as a joint of the robotic limb. This means that each robotic limb has six degrees of freedom. The AstroLimbs can be worn on the astronaut’s backpack, moving and working with the wearer. It acts as a working partner for the wearer during EVA, just like another astronaut. As the outer space environment is almost weightless, the weight and mass of the robotic system will not be applied to the astronaut.

### 2.2. Variable damping control

#### 2.2.1. Model building

**FIGURE 2:**
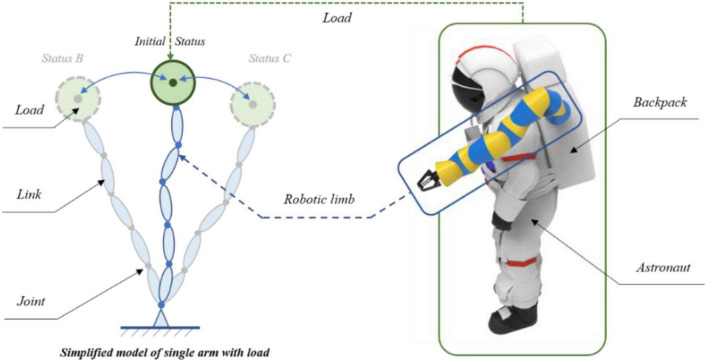
Simplified model of the astronaut and the robotic limb system.

In order to achieve the robotic limb’s ability to maintain the astronaut’s posture during EVA, the variable damping control method based on the Q-learning algorithm was proposed. Prior to the reinforcement learning training, it was necessary to model and simplify the astronaut system with the robotic limbs, which could function faster in the simulation environment, as shown in Figure 2. While the astronaut works outside the ISS cabin, one robotic limb holds the handrail to maintain the position in the working area. Under this condition, the handrail was considered as a fixed end and the end of the robotic limb was simplified to connect to that fixed end. The astronaut and the other robotic limb were combined and simplified into an end-load system, where the second robotic limb mainly provides auxiliary functions, such as tool delivery and operational support. As shown in Figure 2, they were reduced to a green solid ball at the end of the robotic limb. The blue ellipses represent the links of the robotic limb, and these links are connected by rotating joints, which are represented by the solid blue points. Each robotic limb had six degrees of spatial freedom. The fixed end was equal to the handrail of the ISS. The Cartesian coordinate system, which is the absolute coordinate system, was attached to the fixed end. Combined with the forward kinematics of the robotic limb, the end-load movement information for Cartesian space could be obtained in real time.

In addition, this model could also be split into two systems. One was the load system and the other was the robotic limb system without the load. Based on the model, the variable virtual restoring force was introduced to control the load for impact resistance and maintenance of position. In combination with the Q-learning algorithm, the variable damping controller was formed. The virtual restoring force was taken as an external force of the robotic limb. Finally, based on its dynamics, the virtual restoring force could be transformed into the control torque of each joint. In this way, the robotic limb could realize its position-maintaining control to help the astronaut.

#### 2.2.2. Variable damping control for end load

**FIGURE 3:**
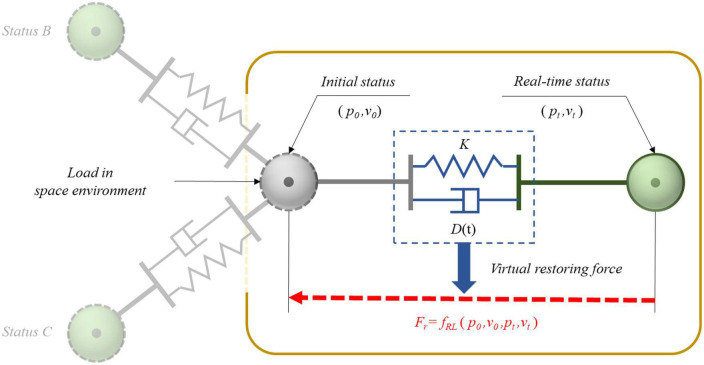
Variable damping control principle for load in weightless space.

In order to achieve the optimum motion characteristics of the robotic limb end after impact, the most straightforward method was to determine the conversion relationship between the motion characteristics of the joint space and the end Cartesian space. It was necessary to discover the configuration changes of the limb in real time and calculate the equivalent moment of each joint inertia. The calculated quantity of the overall process was too high. Thus, the variable damping control method based on the virtual restoring force was introduced. For the load system, it was possible to obtain its absolute movement information in relation to the Cartesian space in real time. In this case, the load could be considered as an unconstrained spatial load that was only controlled by the virtual restoring force, so as to meet the proposed requirements for impact resisting. As shown in Figure 3, the virtual restoring force acted on the mass center of the load, so that the load tended to move back to its original position. Its value varied in real time, which was related to the motion state of the load (*p*_*t*_, *v*_*t*_). The mapping function *f*_*RL*_ between the virtual restoring force and the movement status could be achieved by the Q-learning algorithm.

For the load in weightlessness, in order to reduce the deviation and bring it back to the original position, a virtual restoring force based on the spring damping model was proposed. Its virtual damping coefficient could change adaptively, as shown in Figure 3. The change between the real-time state of the load and the initial state was used as the input of the virtual restoring force, and the virtual restoring force was mainly composed of the virtual spring tension and damping force, which can be shown as follows:


(1) $$F_{r}=K\cdot X\left(t\right)+D\left(t\right)\cdot \dot{X}\left(t\right)$$

where *F*_*r*_ represents the virtual restoring force, *K* is the virtual spring stiffness coefficient, *D*(*t*) is the virtual damping coefficient, *X*(*t*) is the displacement relative to the initial position after impact, and $\dot{X}\left(t\right)$ is the velocity after impact. When the spatial load was impacted in any direction, the corresponding state changes occurred in the three-dimensional space, such as in *Status B* or *C* as shown in Figure 3. The spring damping system was applicable. That is to say, the virtual restoring force generated was always in a straight line with the displacement of the load in relation to the initial state.

For the introduced spring damping system, the corresponding impedance characteristics could be obtained by adjusting the appropriate stiffness coefficient *K* and damping coefficient *D*(t) according to the desired system characteristics. However, the fixed stiffness and damping coefficient could not simultaneously satisfy the overall impact resistance requirements. When the stiffness was fixed, if the damping coefficient was too small, the load-displacement was too large. If the damping coefficient was too large, the recovery speed after impact was too slow. Therefore, the damping coefficient was particularly critical for maximal deviation and recovery time. Considering the practical application of wearable robotic limbs, it was used to hold the handrail of the cabin to stabilize the position of the astronaut when working in a fixed spot. In this case, it was hoped that the equivalent system had a relatively large stiffness. At this time, if the method of variable stiffness was adopted, the stiffness of the system could be reduced, which was not conducive to the astronaut maintaining position. Therefore, the variable damping control method was selected in this paper. For the problem that the virtual restoring force of the fixed damping method could not fully meet the impact resistance requirements, the variable damping controller could change the virtual damping value appropriately depending on the real-time movement state, so as to meet the impact resisting requirements in different states.

#### 2.2.3. Reinforcement learning

When it comes to tackling serialized decision-making issues in unknown contexts, reinforcement learning offers clear advantages. Q-learning is one of the reinforcement learning algorithms and can be used to adaptively learn the virtual damping of load movement in a weightless environment. Therefore, the state of load was divided based on the designed working environment, and the fundamental action was planned. Moreover, the reward function in the task-learning process was proposed.

Reinforcement learning is an overall process that refers to the agent’s trial, evaluation, and action memory (Clifton and Laber, 2020; Chen et al., 2022; Cong et al., 2022; Li et al., 2022). The agent’s learning maps from environment state to action, causing it to reap the greatest rewards after carrying out a particular action. This learning process will make the agent perform best under some preset evaluation rules. The Q-learning algorithm is one of the evaluation rules for the agent to choose a specific action in the present state, which is an action-utility function. Q is short for the word quality, which serves as high-quality feedback for each action and provides the agent with action memory (Ohnishi et al., 2019; Hutabarat et al., 2020). The Q-learning algorithm is excellent for model-free autonomous motion planning when the number of states and actions in the learning process is limited (Clifton and Laber, 2020).

The following equation describes the agent’s corresponding evaluation value after performing the action each time in a particular state:


(2) $$Val=max_{a}Q\left(s,a\right)$$

Where *s* denotes the current state, *a* is the action that can be taken in the current state, and *Val* is the evaluated maximum value corresponding to this action under the circumstances of the current state s and action a. In light of this value, the agent can determine the action to execute in this step.

The core of the Q-learning algorithm is the process of constantly updating the evaluation value *Val* in Equation 2 based on continuous trial training:


(3) $${}_{Q^{\prime }\left(s,a\right)⇐Q\left(s,a\right)+\lambda \left[R\left(s,a\right)+\eta \cdot max_{a^{\prime }}Q\left(s^{\prime },a^{\prime }\right)-Q\left(s,a\right)\right]}$$

where *R* represents the reward value that can be obtained by executing action *a* in the current state *s, s’* is the new state of the agent after executing action *a, a’* is the possible action in state *s’*, λ is the learning efficiency (λ = 0.01), and η serves as the discount factor (η = 0.9).

First, the training was conducted in a single dimension, which simplified the load movement process. Based on the position and velocity information in relation to the Cartesian space, the motion state of the load determined the state of the Q-learning. The following equation provides the definition of the state value:


(4) $${}_{State=f\left(P,Flag\_v\right)}$$

where *State* represents the load motion state, *P* is the displacement compared to its initial position, *Flag_v* denotes the velocity direction identification value depending on both the displacement and velocity direction, which can be expressed as the following Equation 5:


(5) $${}_{Flag\_v=\left\{ \begin{array}{cc} 1 & \vec{P}\cdot \vec{V}\geq 0\\ 0 & \vec{P}\cdot \vec{V}<0 \end{array}\right.}$$

where $\vec{P}$ is the real-time displacement vector, $\vec{V}$ is the real-time speed vector.

In order to improve the efficiency of reinforcement learning, the displacement range was discretized. To guarantee applicability, displacement values outside the valid range were incorporated into adjacent state intervals. And the corresponding relationship between the acquired state and the movement state of the load is shown in Equation 6:


(6) $${}_{State=\left(\left\lceil P/d\right\rceil +s_{int}\right)+n\cdot Flag\_\nu }$$

where *d* is the interval step size for displacement range, *s*_*int*_ is the state offset value designed to count state values from zero, and *n* represents the total number of states regardless of velocity direction. ⌈P/d⌉ stands for the result of rounding up the ratio of *P* to *d*, which is the smallest integer greater than the ratio.

As the load had no gravity in a weightless environment, the control model could be equivalent to a spring-damping model. The force generated by the virtual spring and damping directly acted on the mass center of the load, so the virtual force generated by the real-time virtual spring tension and damping force after impact could be obtained. Thus, the load system’s stiffness-damping characteristics were simulated to achieve optimal motion control. According to the simplified model, the virtual stiffness was designed to be a fixed value, so that the virtual restoring force of the load was proportional to its displacement value.

To avoid excessive displacement, oscillation, and failure to return to its original position after impact, it was necessary to change the virtual damping according to different states. Using the same discrete design idea as the motion state, the maximum virtual damping value was designed to be 600 and the interval step size was 150. Thus, the damping value could be used as an optional action in the Q-learning process in five cases, as shown in Equation 7:


(7) $${}_{Action=\left\{0,150,300,450,600\right\}}$$

During the training process, the agent received a reward for each episode in which they interacted with the environment. For the process that the load suffered an impact in weightless space, it deviated from the original position. Under the action of the virtue spring tension and damping force, it could return to the original position after reaching the maximum deviation. According to the desired impact resistance requirements, the farther the load deviated from the initial position, the weaker its ability was to prevent oscillation, and fewer rewards were given. If the load got closer to the initial position, it obtained more rewards. Therefore, it made sense to take the negative value of the deviation distance as the reward, which can be expressed by the following equation:


(8) $${}_{R=-dis=-\sqrt{x_{t}^{2}+y_{t}^{2}+z_{t}^{2}}}$$

where *R* is the reward value received for a particular action, *dis* is the distance value in relation to the starting position, and *x_*t*_, y_*t*_, and z_*t*_* are the components of the real-time position.

In the training process, when the robot was in the initial position, the reward *R* obtained by the agent was zero. As the reward value *R* was designed to be non-positive, it meant that the reward value of the agent was the maximum in the initial state. After suffering an impact, the robot generated a position deviation. The reward value decreased as the deviation increased. It meant that the agent was punished.

#### 2.2.4. Application on robotic limbs

The impact force applied on an astronaut outside the space station is three-dimensional and can come from any direction. As a result, the displacement and velocity directions of the load do not lie in a uniform line with respect to its initial state. Then the one-dimensional variable damping control method based on the Q-learning algorithm could not completely solve the issue. As the displacement and velocity directions were not in a straight line, a velocity vector was generated in the direction perpendicular to the displacement vector. The system eventually reached equilibrium as a result of the virtual restoring force acting in the displacement direction. The load then moved uniformly around the initial position, and the virtual restoring force provided the load with centripetal acceleration. However, the load was not able to return to the initial position.

**FIGURE 4:**
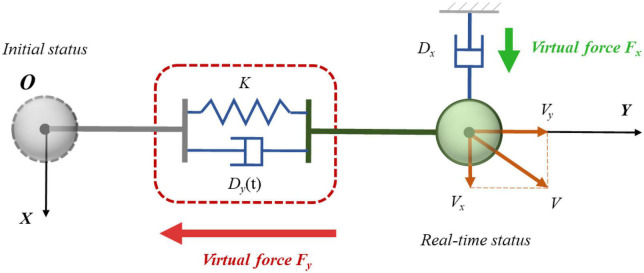
Schematic diagram of three-dimensional impact resistance of the load.

In order to solve the above issue, a speed-decoupling control method based on one-dimensional control was purposed. The improved control principle is shown in Figure 4. The overall concept of this method was to carry out adaptive control in different dimensions through orthogonal decoupling of velocity. In Figure 4, the gray sphere represents the initial state of the load, and the green sphere represents the real-time movement status after impact. The line between the two states is the displacement direction, which was recorded as the *Y* direction. The positive *Y* direction pointed to the direction away from the initial position. The direction perpendicular to the *Y* direction was marked as the *X* direction. The selection of positive *X* direction is shown in Figure 4, which made no difference to the outcome. Since the motion state of the load after impact changed in real time, the *X* and *Y* directions also varied continuously. However, the *Y* direction could be uniquely determined depending on the displacement direction. When the *Y* direction was fixed, the *X* direction then became uniquely determined. The two directions could be determined at any time, even though they were constantly varying in real time. These two real-time directions were the base for orthogonal decoupling velocity. It can be seen from Figure 4 that the load speed *V* was orthogonally decoupled along the *X* and *Y* directions to obtain the velocity component *V*_*x*_ and *V*_*y*_, respectively. The *Y* direction was the key direction for the load to return to the original position after suffering an impact. It was hoped that the load could resist impact in this direction. Therefore, the variable damping controller based on the reinforcement learning method was adopted in the *Y* direction. When *V*_*x*_ became zero, the issue normally transformed into the fundamental problem of impact resisting control for a single direction. Therefore, the control method in this direction was relatively simple, that is to set a large fixed damping coefficient. The velocity in this direction could be quickly reduced to zero as soon as possible.

For the unconstrained load model, the real-time restoring force was virtual and this hypothetical force in the simulation environment had no actual force application object. The load model and the robotic limb model were combined using this virtual force as a bridge. In order to ensure the robotic limb end had the same impact resistance performance as the load model, the force application object of the virtual restoring force should be the robotic limb itself. Hence, the problem was changed into the end force control issue of the series manipulator with six degrees of freedom. In combination with the dynamics of the robotic limb, the joint control torque for the real-time virtual restoring force could be obtained, so as to realize the impact resistance ability of the end load.

The magnitude of the virtual restoring force used to control the end load was in relation to the real-time motion state of the end load, as shown in Equation 9:


(9) $${}_{F_{r}=f_{RL}\left(p_{0},v_{0},p_{t},v_{t}\right)}$$

where *F*_*r*_ is the virtual restoring force acting on the rigid end of the robotic limb, *p*_0_ is the initial displacement, *v*_0_ is the initial velocity, *p*_*t*_ is the real-time displacement after impact, and *v*_*t*_ is the real-time velocity after impact.

Finally, the virtual restoring force of the robotic limb end was brought into the dynamic equation, so that the joint space control torque could be obtained and the impact-resisting control of the robotic limb end could be realized.

**FIGURE 5:**
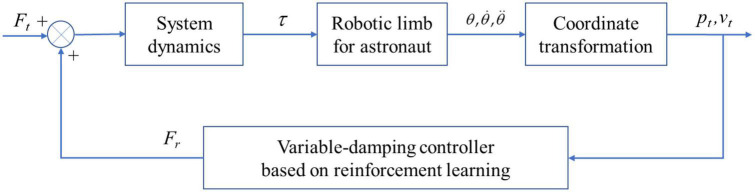
Framework of the variable-damping control method.

A framework of the variable damping control method to further explain the control method is shown in Figure 5. The combined system of an astronaut with a robot was modeled and simplified. With the help of system dynamics and coordinate transformation, the controller enabled the robotic limb end to resist impact. According to Figure 5, *F*_*t*_ stands for the impact applied on the system. *F*_*r*_ is the virtual restoring force originating from the variable damping controller based on the reinforcement learning method. τ is a six-dimensional vector, which stands for the torque of each joint. $\theta ,\dot{\theta },\ddot{\theta }$ is the motion information of each joint. *p*_*t*_ and *v*_*t*_ are the displacement and velocity of the robotic limb end, respectively.

Considering the practical application of wearable robotic limbs, they were used to hold the handrail of the cabins to stabilize the position of the astronaut when working in a fixed spot. In this case, it was hoped that the equivalent system of the robotic limbs and the astronaut had a relatively significant stiffness. At this time, if the method of variable stiffness was adopted, the stiffness of the system would be reduced, which would not be conducive to the astronaut maintaining position. Therefore, the variable damping control method was selected in this paper.

The core of the variable damping controller was that there was always a damping term in the system, and the damping coefficient could be appropriately changed according to the motion effect produced by the external impact. In addition, as the system deviated from its original position, the damping coefficient increased to prevent the system from oscillating. This paper focused on the impact force during a short period and recognized that the system was not subjected to a continuous force. When the damping term of the system persisted, the system eventually became stable.

## 3. Results

### 3.1. Reinforcement learning results

A simulation environment of the unconstrained load was built using the program Virtual Reality Educational Pathfinders (VREP) (Rohmer et al., 2013). The gravity acceleration in the vertical Z direction was set to zero to simulate the outer space environment. Taking the absolute coordinate system of the simulation environment as the reference coordinate system of the load, the real-time movement state could be obtained directly. In this simulation, the load mass was set to 64 kg, the impact force was set to 100N, and its duration time was 500 ms. The force was set to be along the positive direction of the *Y* axis, which acted on the load centroid. The training time for reinforcement learning was designed at 2.5 s so that the load could complete the whole process. The initial moment of the load was in a static state, then it moved in response to an external impact. The corresponding motion state was recorded in real time to obtain the current training state. The next action was selected according to the present state. The agent received a reward according to Equation 8 after each step. The total reward accumulated was recorded in one episode. The whole process was set at 3,000 training times.

**FIGURE 6:**
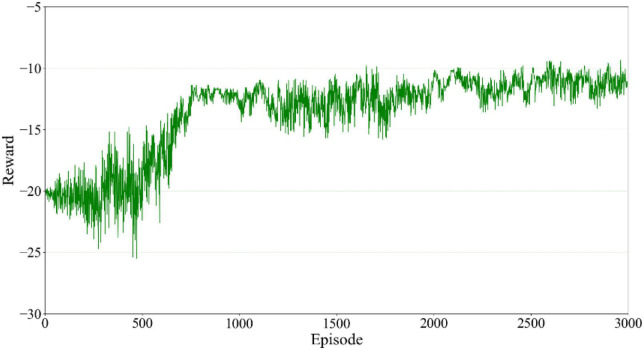
Reward value of each episode for the agent.

The accumulated training reward of each episode is shown in Figure 6. The abscissa is the episode number, and the ordinate represents the total reward value obtained in each episode. According to Equation 8, the reward mechanism adopts a non-positive value so the total reward will be negative. Since the goal of reinforcement learning was to find the optimal strategy to maximize the cumulative reward value, the training performance improved as the cumulative reward value approached zero. According to Figure 6, it can be seen that the agent was an inexperienced individual during the first 750 training episodes. To learn the virtual restoring force control’s damping coefficient and gain more experience, constant trial and error was required. Although the cumulative reward of each episode at this stage fluctuated significantly, it still showed an increasing trend in general. It could be proved that the agent gained some experience in training and the results moved in the right direction. After 750 episodes, the robot gradually learned the task target and the cumulative reward fluctuated slightly. Since the robot action selection strategy adopted the ε-greedy strategy, it enabled the agent with a certain degree of exploration ability. In this case, although the robot learned the action sequence leading to the task target, it still chose to explore a new action sequence with a certain small probability. It converged in the later stage of training and the cumulative reward value fluctuated slightly, which made no difference in the convergence of the whole training process.

**FIGURE 7:**
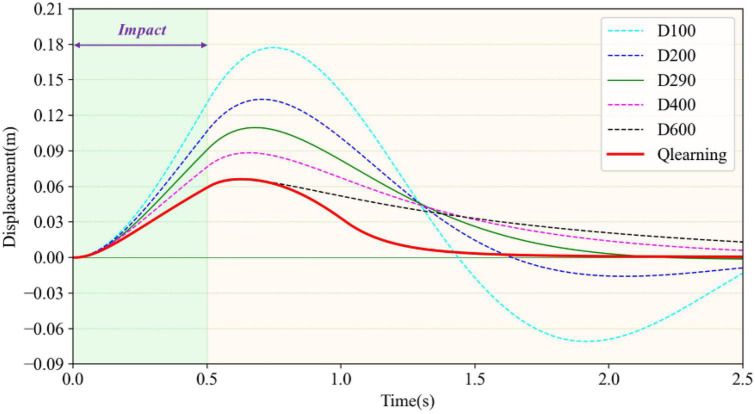
Comparison of load recovery trajectories under different damping values after impact.

The variable-damping controller based on reinforcement learning was tested and the results could be illustrated by the trajectory of the load after impact. The results were compared with fixed damping cases, as shown in Figure 7. The impact force was set as 100N and the duration time was set at 500ms. The stiffness coefficient *K* was set at 500, and the fixed damping coefficient *D* was set at 100, 200, 290, 400, and 600, respectively. That is to say, there were five experiment groups to compare with the reinforcement learning result. As shown in Figure 7, when *D* was 290 as shown by the green solid line, the maximum displacement was 0.11 m and it could return to the initial position within 2.2 s. This value could be seen as the critical virtual damping of the load system. When *D* was 100 or 200, the system was in an underdamped state. It was in the overdamped state when *D* was 400 or 600. In the underdamped state, taking *D* = 100 as an example, shown by the light blue dotted line, the maximum displacement was 0.18 m, which was 0.07 m greater than the maximum displacement of critical damping. It could return to the initial position within 1.5 s. However, the load still had speed and failed to stop. It moved to the reverse maximum position and then moved back. In this way, the oscillation in relation to the initial position occurred repeatedly. Furthermore, it was unable to return to the initial position or stop within 2.5 s. When the load was in the overdamping state, taking D = 600 as an example, shown by the black dotted line, the maximum displacement after impact was 0.065 m, which was less than the maximum displacement of critical damping by 0.045 m and far less than the maximum displacement of under damping by 0.115 m. The load recovered very slowly because of the excessive damping. It moved towards the initial position during the recovery phase, but could not stop at the initial point within the specified time. There was still a position deviation of 0.015 m at 2.5 s.

The trajectory generated by the reinforcement learning algorithm is shown by the red solid line. The portion of the trajectory where the load started to deviate from its initial position after impact completely coincided with the overdamping case (*D* = 600), which indicates that the maximum damping was selected in the early stage to minimize displacement. When the impact weared off, the load began to return to its initial position after reaching maximum displacement under the virtual restoring force. For this process, the damping coefficient of reinforcement learning first decreased and then rose, so that the load could move towards the initial position quickly and try to stop at the initial point without overshooting. It can be seen from the red solid line that the load returned to the initial position within 1.7 s and finally remained stable, indicating the rapidity, stability, and recoverability of impact resistance. The variable damping control method took advantage of the small displacement deviation of the large damping case and the fast return of the small damping case. Compared with the critical damping case (*D* = 290) with better control effect in fixed damping, the maximum displacement of the reinforcement learning method reduced by 40.9% and the time to return to the original position shortened by 22.7%. Therefore, the variable damping control method met the requirements for impact resistance and pose maintenance.

### 3.2. Variable damping control results of end load

In order to evaluate the training results of reinforcement learning and solve the impact resistance problem subjected to three-dimensional impact, relative tests were carried out. According to Figure 4, the variable damping control method based on Q-learning was adopted for the dimension along the displacement direction, recorded as controller *Y*. The fixed damping control method was adopted for the dimension in vertical to displacement direction, recorded as controller *X*. Based on the orthogonal decoupling method for three-dimensional impact, four simulation experiments were designed. In these groups, the damping factor in the direction of vertical displacement *X* was set to a fixed value (*D_*x*_* = 600), and the stiffness coefficient along the direction of displacement *Y* was set to 500. The damping coefficients were selected depending on the underdamping case, critical damping case, overdamping case, and variable-damping case. The corresponding values were recorded as 100, 290, 600, and Q-learning. The damping coefficient of the Q-learning method was variable. In these experiments, the velocity and displacement were not in the same straight line after the three-dimensional impact. Three components of the impact along *YZX* directions were continuously applied to the load within the first 1.5s. Each magnitude of the impact force was set at 100N and the duration time was 500ms. The results of different controllers were compared and analyzed.

**FIGURE 8:**
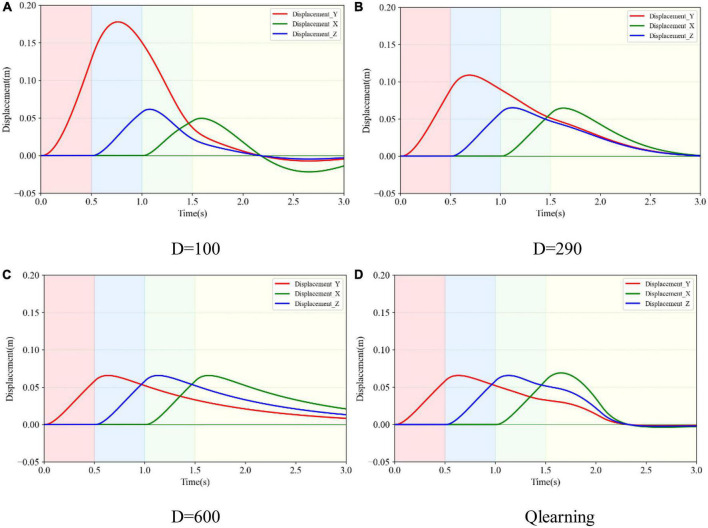
Three-dimensional displacement variance of the load subjected to three-dimensional impact in different damping cases. **(A)** Under damping case. **(B)** Critical damping case. **(C)** Over damping case. **(D)** Variable damping case based on Q-learning algorithm.

Figure 8 indicates the displacement variance in *XYZ* directions after a three-dimensional impact. The solid red, blue, and green lines represent the trajectory changes in *YZX* directions, respectively. Taking Figure 8A as an example, only after the impact force was exerted in the appropriate direction did the corresponding displacement occur. At the starting time, the impact force was applied in the *Y* direction and the corresponding solid red line rose. The impact force in the *Y* direction disappeared after 0.5 s. At the time of 0.5 s, the impact force in the *Z* direction was exerted and disappeared after 0.5 s. The blue solid line kept rising. Similarly, the impact force in the *X* direction was applied during 1.0–1.5s, and the green solid line began to creep up. Comparing Figures 8A–D, it can be seen that the displacement change in the *Y* direction was the largest, of 0.18, 0.11, 0.065, and 0.065 m, respectively. They were consistent with the displacement change of the load after the unidirectional impact. The deviation from the initial position of the variable damping method after suffering an impact was the smallest. Comparing the *X* and *Z* directions, when in underdamping case (*D* = 100), there was an oscillation in the *X* direction. When in overdamping case (*D* = 600), it failed to return to the initial position in both *X* and *Z* directions within 3.0 s. When in the critical damping situation (*D* = 290), it took 3.0 s to return to the initial position. When in the variable damping case (Q-learning), it returned to the starting point within 2.3 s. The variable damping case took the least time to return.

**FIGURE 9:**
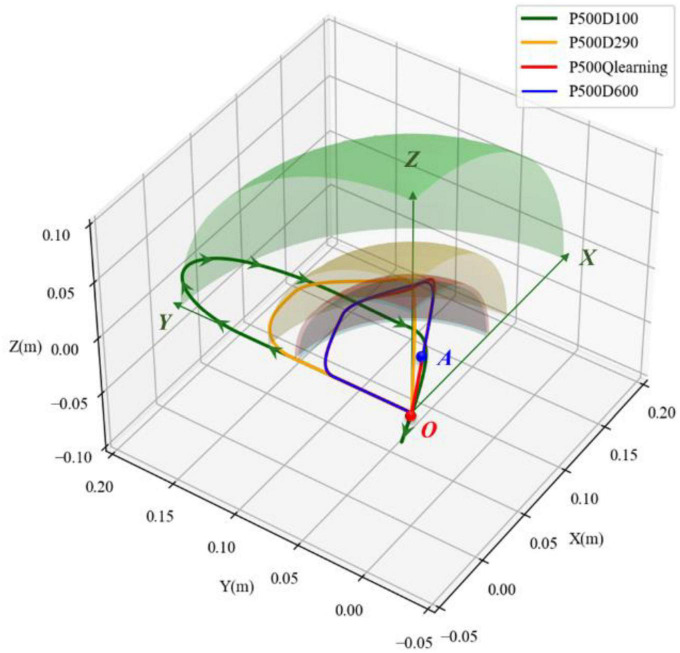
Comparison of spatial movement trajectories in different damping cases.

The space motion trajectories generated by four experimental groups were compared, and the results are illustrated in Figure 9. Point *O* denotes the initial position of the load. The green straight line with an arrow represents the *XYZ* directions. The movement trajectories of underdamping, critical damping, overdamping, and variable damping control method are represented by green, yellow, blue, and red solid lines, respectively. It can be seen that the load was on point *O* at the initial time, and its motion trajectory was an irregular curve in space. From the perspective of Figure 9, the motion direction of the load was basically clockwise, according to the green arrows of the curve. The load first moved along the direction of increasing *Y*, then turned to the direction of increasing *Z*. After that the load moved to the direction of increasing *X*. Finally, it moveD back towards the origin point.

For each of the four motion trajectories, the maximum deviation values in relation to the initial position during the whole process was obtained. Taking this maximum displacement as the radius and the initial position as the center point of a sphere, the spheres with the maximal displacement under different controllers could be obtained. The maximal displacement spheres of the four groups are shown as the transparent surface, respectively, in Figure 9. The colors of these spheres are the same as their motion trajectories. For better comparison, only one-eighth of the maximal displacement sphere for the main motion space is shown. Comparing these transparent-colored surfaces, it could be observed that the smaller the damping factor was selected, the larger the sphere was. The other three groups of maximal displacement spheres were wrapped by the sphere (green transparent sphere) with the underdamping case (*D* = 100). The spheres of the overdamping case (*D* = 600) and variable damping case (Q-learning) almost coincided.

For the motion on the underdamping condition (*D* = 600), it could not stop immediately when the load returned to the initial position, according to its motion trajectory formed by the green solid line. However, it continued to move in the opposite direction through the origin for a certain distance and then returned. It resulted in oscillation in relation to the initial position. This corresponds to the part of the green solid line formed before the original point *O*. Combining the displacement curves in three directions in Figure 8A further supports the existence of oscillation.

For the two conditions of overdamping (*D* = 600) and variable damping (Q-learning), according to Figures 8C, D, the change magnitude and trend of the load-displacement in three directions of *XYZ* within 1.5s were basically the same. However, the load returned to its original position faster on the condition of variable damping. It could quickly return to the initial motion state within 2.3s and remain stable. In contrast, on the condition of overdamping, the load could not even return to the initial point within 3.0s. In terms of fast return, the performance with fixed large damping was not ideal.

The maximal displacement of load and the time taken to return to the original state after three-dimensional impact on four cases were comprehensively compared. The maximum displacement can serve as a good indicator of impact resistance stability. The impact resistance and stability will be greater and better as this index’s value decreases. The time taken to recover to the initial state can be a good indicator of oscillation resistance. The less time required, the more quickly it will return to the initial state and the stronger its resilience will be. The maximal displacement on the variable damping condition (Q-learning) was 0.089m, which was basically consistent with the maximal displacement on the overdamping condition (*D* = 600), which was less than the maximal displacement of 0.18 m and 0.11m in the underdamping case (*D* = 100) and critical damping (*D* = 290) case. The values reduced by 50.6% and 19.1%, respectively, compared to the underdamping and critical damping cases. The performance of the variable damping controller was better. Moreover, the time taken to restore to the initial state under the variable damping condition (Q-learning) was 2.3 s, which was the least time consumed in the four groups. It was less than the counterparts on the critical damping condition (*D* = 290) and the overdamping condition (*D* = 600) with 3.1 and 5.2 s, which reduced by 25.8% and 55.8%. On the condition of underdamping (*D* = 100), the load could not return to its original position or remain stable after impact. In this case, its recovery performance was the worst. Therefore, according to the index comparison of the time taken to return to the initial state, the variable damping controller performed better in terms of a fast return. Based on the comparison results, it can be seen that the system showed the best stability, rapidity, and accuracy after suffering the impact under the variable damping method based on Q-learning, which verifies the feasibility and superiority of this method in impact resistance and position maintenance.

### 3.3. Variable damping control results of the robotic limbs

**FIGURE 10:**
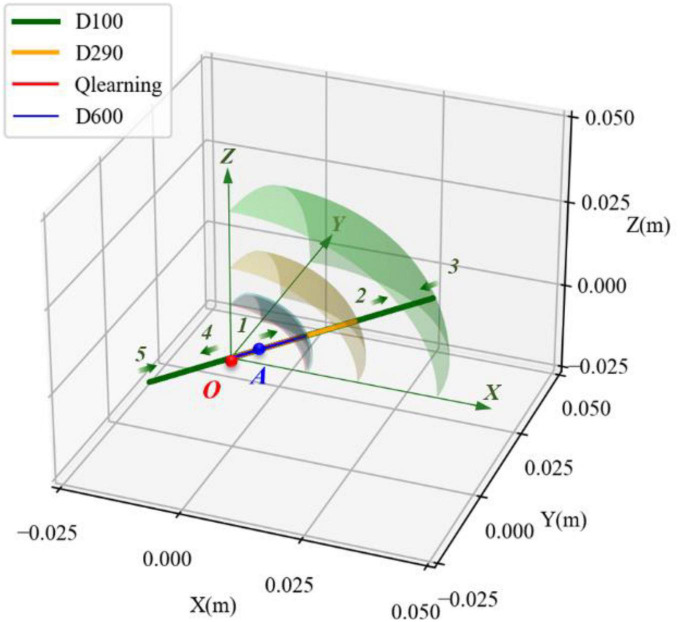
Comparison of motion trajectories of the robotic end in different damping cases.

**FIGURE 11:**
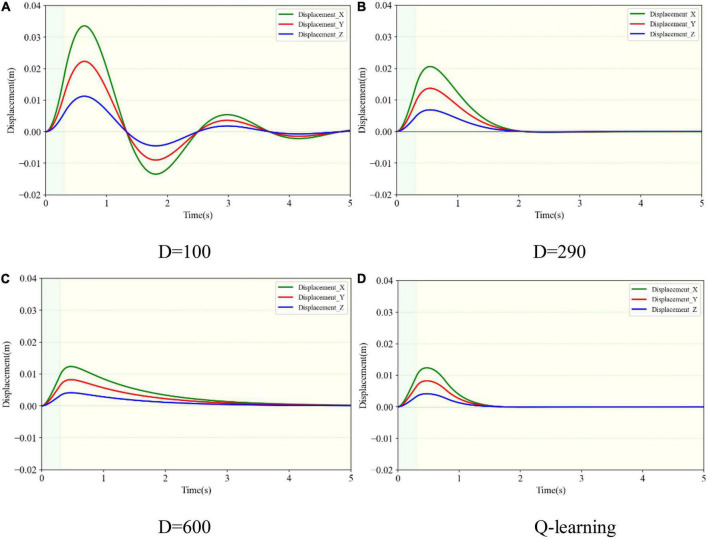
Three-dimensional displacement changes of the robotic end subjected to three-dimensional impact. **(A)** Under damping case. **(B)** Critical damping case. **(C)** Over damping case. **(D)** Variable damping case based on Q-learning algorithm.

The simulation tests for the motion performance of the impact-affected end of the load were carried out in combination with the variable damping method and the dynamics of the robotic limb. The load was the same as in the above tests and connected to the robotic limb’s end to form a system of manipulating the load. The load at the end of the robotic limb was subjected to external impact. The force acted on the load centroid, whose components in the *XYZ* axes were designed to be 30, 20, and 10 N. The duration time was set at 300 ms. Based on the simulation conditions, the motion performances of the robotic limb’s end under four different damping controllers were compared. The experimental groups included three fixed damping cases (D = 100, 290, and 600) and one variable damping test based on the Q-learning algorithm. The four motion trajectories and the projections in the *XYZ* directions of the end load with respect to its initial position after impact are shown in Figures 10, 11.

The trajectories corresponding to the four simulation conditions were colored green, yellow, blue, and red. The origin *O* of the coordinate system in the figure represents the initial position of the end load, and the green straight lines with arrows represent the *XYZ* directions. They were consistent with the directions of the spatial absolute coordinate system. As shown in Figure 10, the end load started to move in the impact direction of the green arrows after the external impact force. Under the action of the restoring force, it moved toward the initial position after reaching the maximum displacement. Due to the underdamping case, the end load did not directly stop at the initial position. However, it moved past the initial position first and then returned, resulting in oscillation relative to the initial position. Its movement sequence is shown as the serial number from 1 to 5 in Figure 10. Yet on the other three conditions, the end load did not oscillate when receiving the impact force.

The maximal displacement of the end load under different conditions could be obtained in the same way as in section 3.2. The envelope surface of the maximum displacement of the end load is depicted in Figure 9 as the transparent surface. The maximal displacement in the underdamping case (*D* = 100) was 0.042 m, which was the largest. The counterpart in the variable damping case (Q-learning) was 0.015 m and it was the smallest. By contrast, the maximal displacement in the variable damping case was reduced by 64.3%. As shown in Figure 11, for the variable damping method, the load could restore to the initial state faster without oscillation, in a time of 1.65 s. However, for the condition of overdamping, the end load could not return to the initial position within 1.65s and only moved to point *A*. At this time, the distance between points *O* and *A* was 0.006 m, accounting for 40.0% of the maximal displacement in the whole process. Although the maximal displacement of the end load for the overdamping test was the least, its ability to return to the starting position was not strong. Compared with the underdamping and overdamping cases, the maximal displacement value and recovery time results of the critical damping case fell somewhere in between. For the case of underdamping, oscillation occurred and the load could not return to the initial position within 5 s, which was the maximal time designed for one single simulation episode. Therefore, it was considered that the recovery time was too long to meet the requirement for fast return, and the corresponding indicators were not compared. Thus, comparing the recovery time of the critical damping and variable damping cases, the former took 2.05 s and the latter only needed 1.65 s. The variable damping’s recovery time was cut by 19.5%.

**FIGURE 12:**
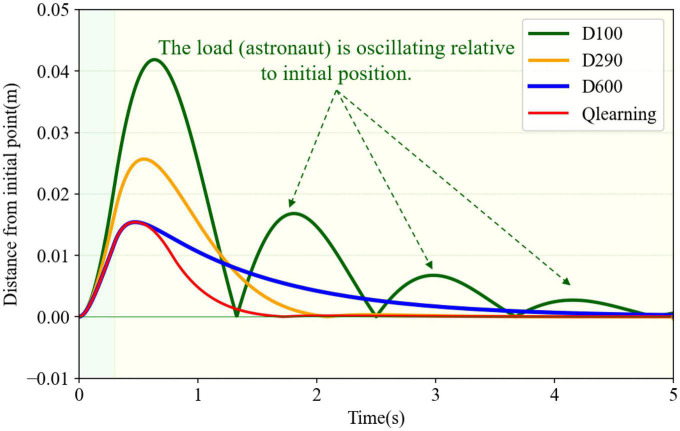
Distance from the initial point in different damping cases.

In order to compare the change of spatial distance with time between the real-time position and its initial point. Distance from the initial point under four different damping cases are shown in Figure 12, whose values were calculated by Equation 8. The last three conditions had a similar varying trend of distance, which first increased and then reduced to zero. However, in the underdamping case (*D* = 100), the trend changed periodically with amplitude attenuation. The load oscillated relative to its initial position on this condition. Compared to the other three cases, the variable damping method had the minimal deviation distance and the shortest return time. Furthermore, it could return directly to the initial position without oscillation.

In terms of resistance to impact at the end of the robotic limb, based on the above analysis, the end load with variable damping controller based on the Q-learning algorithm could quickly return to the initial position and stop after impact. During this process, the variable damping case had the least maximal displacement and minimal recovery time. It enabled the robotic limb to return fast and prevent oscillation.

**FIGURE 13:**
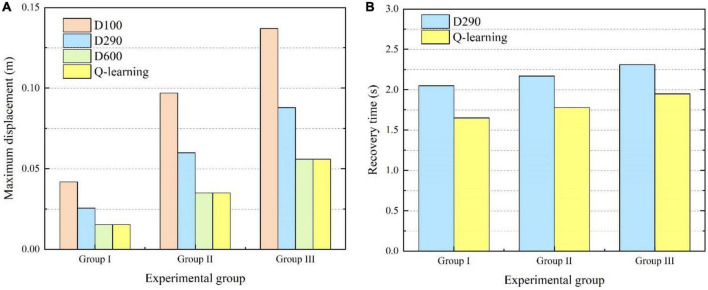
Comparisons of maximum displacement and recovery time for different experimental groups. **(A)** Comparison of maximum displacement. **(B)** Comparison of recovery time.

In order to further verify the effectiveness of the proposed variable damping controller, experiments were carried out for different external impacts. For experimental group I, the force components in the *XYZ* axes were designed to be 30, 20, and 10 N. The duration time was set as 300 ms. For experimental group II, the force components in the *XYZ* axes were designed to be 50, 40, and 30 N. The duration time was set as 400 ms. For experimental group III, the force components in the *XYZ* axes were designed to be 50, 50, and 50 N. The duration time was set as 500 ms. Thus, the total impulse of external impact in the three experiments was 11.2, 28.3, and 43.3 Ns. Four different damping control methods were tested in each experimental group. The maximum displacement from the original point and recovery time in each case were emphatically compared and analyzed, as shown in Figure 13. As shown in Figure 13A, it can be seen that the system’s maximum displacement was the least by the variable damping method for the three different impacts. Compared with the maximum displacement values to the underdamping and critical damping cases of all three experimental groups, the variable damping system’s values were reduced by 39.3% and 62.1% on average. The underdamping system oscillated and the overdamping system could not stop within the specified time. Thus, for the recovery time, only the critical damping and variable damping were compared, as shown in Figure 13B. Compared with the critical damping method for the three different impacts, the variable damping method’s return time was cut by 17.7% on average.

## 4. Discussion

This paper studied the issue of providing impact-resisting and position maintaining assistance for astronauts during EVA without the help of the space station’s robotic arms. A wearable robotic limb system was introduced to give astronauts extra arms, which could help resist impact and maintain their position during EVA. The impact-resisting requirements for astronauts during EVA were analyzed. A variable damping controller based on the reinforcement learning algorithm was proposed. The combination system of an astronaut with robotic limbs was modeled and simplified. Compared with the fixed damping control method, the variable damping control method could meet all the impact-resisting requirements well by itself. It had better performance in preventing excessive deviation and exhibited fast return to the starting point. Meanwhile, it also had the capability of preventing oscillation and returning to the original position accurately. In the end, the appropriate simulation environment was built, and simulation experiments were conducted to confirm the method’s rationality and viability.

However, there are still some limitations of the proposed method that will affect the performance in real-world situation. First, the weight of the astronaut and backpack was regarded as unchangeable in the simulation process. However, the actual situation is that for different astronauts, this value would slightly change. In order to improve the applicability of the method, this parameter also needs be taken as the input of the algorithm in further research. Second, the simulation environment was used for method validation, which is different from the real environment. It limits the experimental tests of the proposed method in practical application. In the future, it is necessary to set up a weightless experimental platform on the ground to simulate the outer space environment. We should let wearers with different weights carry out the relative tests to further verify the feasibility of the proposed method.

## Data availability statement

The original contributions presented in this study are included in the article/Supplementary material, further inquiries can be directed to the corresponding author.

## Author contributions

SZ and YZ proposed the concept. SZ, TZ, and DS established the algorithm model and carried out the simulation experiments under the supervision of JZ and YZ. SZ wrote the original manuscript. TZ and DS helped review the manuscript. All authors read and agreed on the final version of the manuscript.
